# Supplementary material for: Modulating PKCα Activity to Target Wnt/β-Catenin Signaling in Colon Cancer
Source: Cancers (Basel). 2019 May 18;11(5):693. doi: 10.3390/cancers11050693 (PMC6563011; doi:10.3390/cancers11050693)
Supplement: Supplementary file 1 [file cancers-11-00693-s001.pdf]

Supplementary Materials: Modulating PKCα Activity to Target Wnt/β-Catenin Signaling in Colon Cancer

Sébastien Dupasquier, Philippe Blache, Laurence Picque Lasorsa, Han Zhao, Jean-Daniel Abraham, Jody J. Haigh, Marc Ychou and Corinne Prévostel

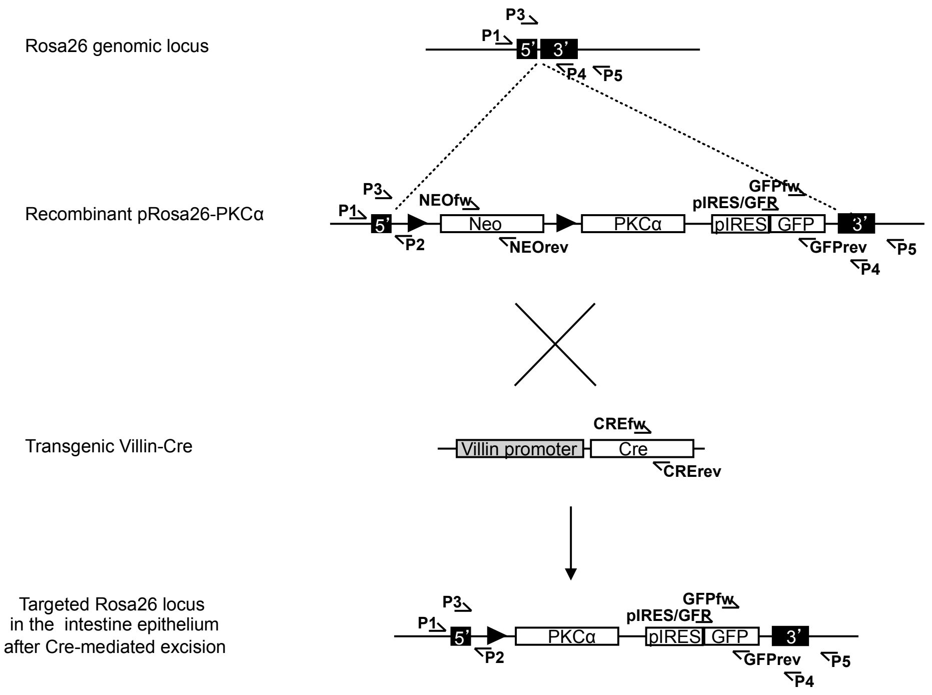

Figure S1. Recombination strategy to induce PKCα overexpression specifically in the intestinal epithelium. The primers used to screen *PRCKA* cDNA knock-in mice and villin-Cre mice and the size of the resulting PCR amplicons are listed in Table S4.

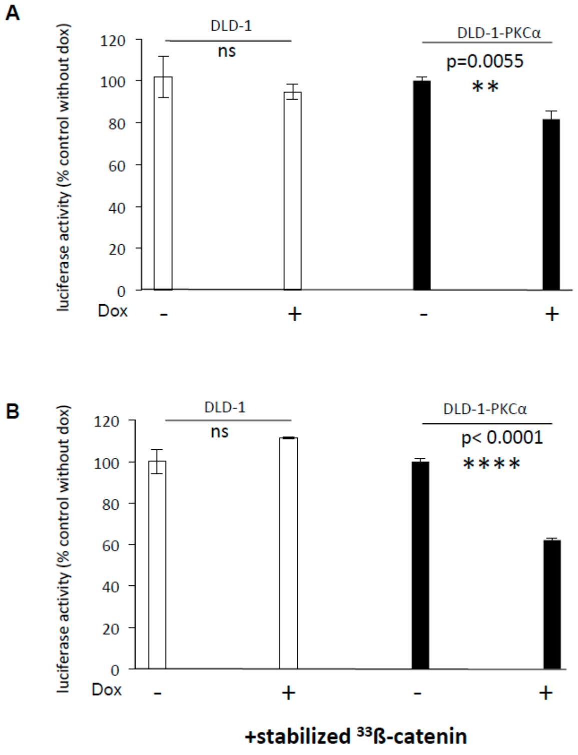

Figure S2. TOPflash reporter luciferase assays showing the inhibition of the Wnt/β-catenin signaling pathway activity upon incubation with doxycycline and PMA in A. DLD-1-PKCα cells (doxycycline-inducible PKCα expression) and B. DLD-1-PKCα cells that overexpress stabilized β-catenin.

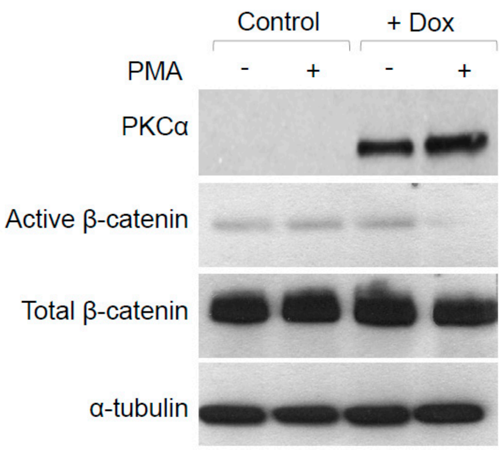

Figure S3. Western blot showing the decrease of active β-catenin upon incubation with both doxycycline and PMA-induced PKCα expression (see upper panel) and activation respectively versus total β-catenin and α-tubulin (loading control) in whole extracts of DLD-1-PKCα cells.

Table S1. PKCα mutations identified in human intestinal tumors (http://cancer.sanger.ac.uk/cosmic).

Sample Name	Sample ID	AA Mutation	CDS Mutation	Primary Tissue	Tissue Subtype	Tissue Subtype	Histology	Histology Subtype	Somatic Status	Sample Source	Zygosity
HT115	2301987	p.A17T	c.49G>A	Large intestine	NS	NS	Carcinoma	Adenocarcinoma	Variant of unknown origin	Cultured	Heterozygous
HT115	2301987	p.A25A	c.75G>A	Large intestine	NS	NS	Carcinoma	Adenocarcinoma	Variant of unknown origin	Cultured	Heterozygous
61	2497169	p.V31M	c.91G>A	Small intestine	Duodenum	NS	Adenoma	NS	Confirmed Somatic	Tumour Sample	Unknown
PD1519a	753434	p.P98S	c.292C>T	Large intestine	NS	NS	Carcinoma	Adenocarcinoma	Confirmed Somatic	Tumour Sample	Heterozygous
TCGA-G4-6588-01	1651337	p.R99fs*32	c.291delC	Large intestine	Caecum	NS	Carcinoma	Adenocarcinoma	Confirmed Somatic	Unknown	Unknown
TCGA-AD-6889-01	1651142	p.L121P	c.362T>C	Large intestine	Colon	Ascending	Carcinoma	Adenocarcinoma	Confirmed Somatic	Unknown	Unknown
TCGA-G4-6306-01	1651324	p.C135C	c.405C>T	Large intestine	Colon	Ascending	Carcinoma	Adenocarcinoma	Confirmed Somatic	Unknown	Unknown
CRC-03T	2456774	p.C143C	c.429C>T	Large intestine	NS	NS	Carcinoma	Adenocarcinoma	Confirmed Somatic	Tumour Sample	Unknown
LS411	2301999	p.K172K	c.516G>A	Large intestine	Colon	Right	Carcinoma	Adenocarcinoma	Variant of unknown origin	Cultured	Heterozygous
HUB-02-B2-113	2607139	p.N182fs*2	c.540delA	Large intestine	NS	NS	Carcinoma	Adenocarcinoma	Confirmed Somatic	Unknown	Unknown
HUB-02-B2-120	2607144	p.N182fs*2	c.540delA	Large intestine	NS	NS	Carcinoma	Adenocarcinoma	Confirmed Somatic	Unknown	Unknown
HCC2998	2301977	p.L231L	c.691T>C	Large intestine	NS	NS	Carcinoma	Adenocarcinoma	Variant of unknown origin	Cultured	Heterozygous
SNU-175	2302007	p.T337T	c.1011G>A	Large intestine	NS	NS	Carcinoma	Adenocarcinoma	Variant of unknown origin	Cultured	Heterozygous
T2991	2296153	p.D338Y	c.1012G>T	Large intestine	Colon	Transverse	Carcinoma	Adenocarcinoma	Confirmed Somatic	Unknown	Unknown
HCC2998	2301977	p.F341F	c.1023C>T	Large intestine	NS	NS	Carcinoma	Adenocarcinoma	Variant of unknown origin	Cultured	Heterozygous
CRC-19T	2456792	p.D357N	c.1069G>A	Large intestine	NS	NS	Carcinoma	Adenocarcinoma	Confirmed Somatic	Tumour Sample	Unknown
TCGA-F5-6814-01	1651640	p.I367I	c.1101C>A	Large intestine	Rectum	NS	Carcinoma	Adenocarcinoma	Confirmed Somatic	Tumour Sample	Unknown
T3144	2296185	p.V381M	c.1141G>A	Large intestine	Rectum	NS	Carcinoma	Adenocarcinoma	Confirmed Somatic	Unknown	Unknown
C108	2293713	p.M417I	c.1251G>A	Large intestine	NS	NS	Carcinoma	Adenocarcinoma	Confirmed Somatic	Unknown	Heterozygous
sysucc-311T	2456736	p.A444V	c.1331C>T	Large intestine	NS	NS	Carcinoma	Adenocarcinoma	Confirmed Somatic	Tumour Sample	Unknown
T613	2296257	p.A444A	c.1332G>A	Large intestine	Caecum	NS	Carcinoma	Adenocarcinoma	Confirmed Somatic	Unknown	Unknown
Gp5D	2301974	p.V469A	c.1406T>C	Large intestine	NS	NS	Carcinoma	Adenocarcinoma	Variant of unknown origin	Cultured	Heterozygous
Gp2D	2301973	p.V469A	c.1406T>C	Large intestine	NS	NS	Carcinoma	Adenocarcinoma	Variant of unknown origin	Cultured	Homozygous
63	2497170	p.H476R	c.1427A>G	Small intestine	Duodenum	NS	Adenoma	NS	Confirmed Somatic	Tumour Sample	Unknown
TCGA-AA-A010-01	1651109	p.D481E	c.1443C>A	Large intestine	Colon	Transverse	Carcinoma	Adenocarcinoma	Confirmed Somatic	Unknown	Unknown
SW48	2302017	p.K486N	c.1458G>T	Large intestine	NS	NS	Carcinoma	Adenocarcinoma	Variant of unknown origin	Cultured	Heterozygous
TCGA-A6-6141-01	1650953	p.I505I	c.1515C>T	Large intestine	Caecum	NS	Carcinoma	Adenocarcinoma	Confirmed Somatic	Unknown	Unknown
sysucc-311T	2456736	p.A506T	c.1516G>A	Large intestine	NS	NS	Carcinoma	Adenocarcinoma	Confirmed Somatic	Tumour Sample	Unknown
LS174T	2301997	p.G534R	c.1600G>A	Large intestine	NS	NS	Carcinoma	Adenocarcinoma	Variant of unknown origin	Cultured	Heterozygous
LS180	2301998	p.G534R	c.1600G>A	Large intestine	NS	NS	Carcinoma	Adenocarcinoma	Variant of unknown origin	Cultured	Heterozygous
HCT116	2301978	p.A578V	c.1733C>T	Large intestine	NS	NS	Carcinoma	Adenocarcinoma	Variant of unknown origin	Cultured	Heterozygous
TCGA-AA-3864-01	1651060	p.L581L	c.1741C>T	Large intestine	Caecum	NS	Carcinoma	Adenocarcinoma	Confirmed Somatic	Unknown	Unknown
T1154	2296082	p.D590D	c.1770C>T	Large intestine	Colon	Transverse	Carcinoma	Adenocarcinoma	Confirmed Somatic	Unknown	Unknown
CCK81	2301965	p.R598W	c.1792C>T	Large intestine	NS	NS	Carcinoma	Adenocarcinoma	Variant of unknown origin	Cultured	Heterozygous
6948_PT	2500976	p.R632*	c.1894C>T	Large intestine	Colon	Left	Carcinoma	Adenocarcinoma	Confirmed Somatic	Tumour Sample	Unknown
6948_CLM	2500977	p.R632*	c.1894C>T	Large intestine	Colon	Left	Carcinoma	Adenocarcinoma	Confirmed Somatic	Tumour Sample	Unknown
Gp5D	2301974	p.Y658C	c.1973A>G	Large intestine	NS	NS	Carcinoma	Adenocarcinoma	Variant of unknown origin	Cultured	Heterozygous
Gp2D	2301973	p.Y658C	c.1973A>G	Large intestine	NS	NS	Carcinoma	Adenocarcinoma	Variant of unknown origin	Cultured	Homozygous
SW948	2302021	p.A671A	c.2013A>G	Large intestine	NS	NS	Carcinoma	Adenocarcinoma	Variant of unknown origin	Cultured	Homozygous

	Silent mutations
	Non silent mutations located in variable (non conserved) domains
	Non silent mutations located in the conserved DAG/Phorbol esters binding sites (C1A and C1B)
	Non silent mutations located in the conserved calcium binding site (C2)
	Non silent mutations located in the conserved kinase domain

Table S2. Sequence variations identified by sequencing the PKCα coding sequence (PRKCA gene exons) of the eight CRC cell lines used in the study.

Cell line	Change (CDS)	Change (AA)	Zygosity	GenBank
DLD-1	c.897 G>A	p. M299I	heterozygous	
HCT116	c.1779 G>A	p. E593E	heterozygous	XM_004041114.2 *Gorilla*
RKO	c.1779 G>A	p. E593E	heterozygous	XM_004041114.2 *Gorilla*
HT29	c.831 G>A	p. L277L	heterozygous	AB527684.1

Table S3. Primers used for PCR amplification and sequencing of the PKCα coding sequence (PRKCA exons) of the eight CRC cell lines used in the study.

PRKCA Exon	Forward Primer	Reverse Primer
1	GGAGGCAAGAGGTGGTTGG	GCGATGAATTTGTGGTCCTT
1	GGACCATGGCTGACGTTTT	GGTTCCAAGTTATCGGAGTGAG
2	TCATTGGGTTTCACATACAAACCTT	GCCCAAATTTACAGCCCTCATA
3	GAAATGCCTTCTGATTGCTG	CAGTTGCACAGGCGTTAAAA
4	GCCTGAGCTTGGCTTAATCT	TTACTGAGCCTTCCTTGCTCA
5	TGCACCAAAACACTGAGGAG	CAGTGTAACCTTCATCCAAATGC
6	GACAGCGGGCAATATAGGTC	GGCAACTGCATGGTGTATAGG
7	ACATGTGGTATCTCCCAAGAAA	AATGGTCTGATGCTTTGAGACA
8	TTCACAAAACCGCTCGACTA	TTCCTGAAGTTGGGCTTTCT
9	CAGAAAAATGACCCACGTGTT	AGGAGAACAAAACTGATTCGACA
10	CCCCCAAGATATGTGCTTACA	TGCTAAGAAGCTTTGGGCTGA
11	AGAGCAAAGGAAGCCACTTG	TGGTAGGACTTGGTTGTTCAAA
12	GGCATCTAAGGAAGCAAGTGA	GAGGCCCAGCTAACCTCTCT
13	CAGTTCCAAAGCAAACCATGT	GAGAGGGCTGCGTTTACAGT
14	TGCCTACCTGCTTGACTTACC	ACGTCAGGTCTTGCTCTCG
15	CAGGCACCATGTGAATGAAT	GCTCTTGGTCCATGATCACA
16	GCAGAGCTAGGCAAATGGAA	CTCCCAGAATTTGGGGACAT
17	GAGAGCTGCTCCCGCATT	GGCTGGGGAGGTGTTTGT

Table S4. Primers used for screening 129/Sv ES cells, PKCα knock-in mice and villin-Cre transgenic mice. PKCαKI = ES cell clones containing one PRKCA-KI recombinant allele within the Rosa26 locus; PKCαKI = ES cell clones containing two PRKCA-KI recombinant alleles within the Rosa26 locus; PKCαKI-Villin-Cre = ES cell clones containing both one PRKCA-KI recombinant alleles within the Rosa26 locus and the Villin-Cre transgene; PKCαKIKI-Villin-Cre = ES cell clones containing both two PRKCA-KI recombinant alleles within the Rosa26 locus and the Villin-Cre transgene.

Primer	Sequence	Amplicon Size				
**Name**	**5′->3′**	**WT**	**PKCαKI**	**PKCαKIKI**	**PKCαKI /Villin-Cre**	**PKCαKIKI /Villin-Cre**
P1	TAGGTAGGGGATCGGGACTCT	1253	1253	1253	1253	1253
P2	GCGAAGAGTTTGTCCTCAACC					
P3	AAAGTCGCTCTGAGTTGTTAT	602	602		602	
P4	GGAGCGGGAGAAATGGATATG					
GFPfw	CTCTCGGCATGGACGAGCTG		4765	4765	4765	4765
P5	CAGTGGCTCAACAACACTTGGTC					
pIRES-GFP	GGCCACAACCATGGTGAGCAA		390	390	390	390
GFPrev	CTTCAGCTCGATGCGGTTCACC					
NEOfw	GGAAGGGACTGGCTGCTATTG		487	487	487	487
NEOrev	CGATACCGTAAAGCACGAGG					
NEOfw	CAAGCCTGGCTCGACGGCC				198	198
NEOrev	CGCGAACATCTTCAGGTTCT					
